# Supplementary material for: Causal interplay between lactose intolerance and gut microbiota: a combined bidirectional Mendelian randomization and in vivo validation study
Source: Front Nutr. 2026 Jun 1;13:1803337. doi: 10.3389/fnut.2026.1803337 (PMC13265576; doi:10.3389/fnut.2026.1803337)
Supplement: Supplementary file 1 [file Data_Sheet_1.zip › supplementary materials/Forward/forest plot/ebi-a-GCST90027649.finngen_R12_E4_LACTONAS.pdf]

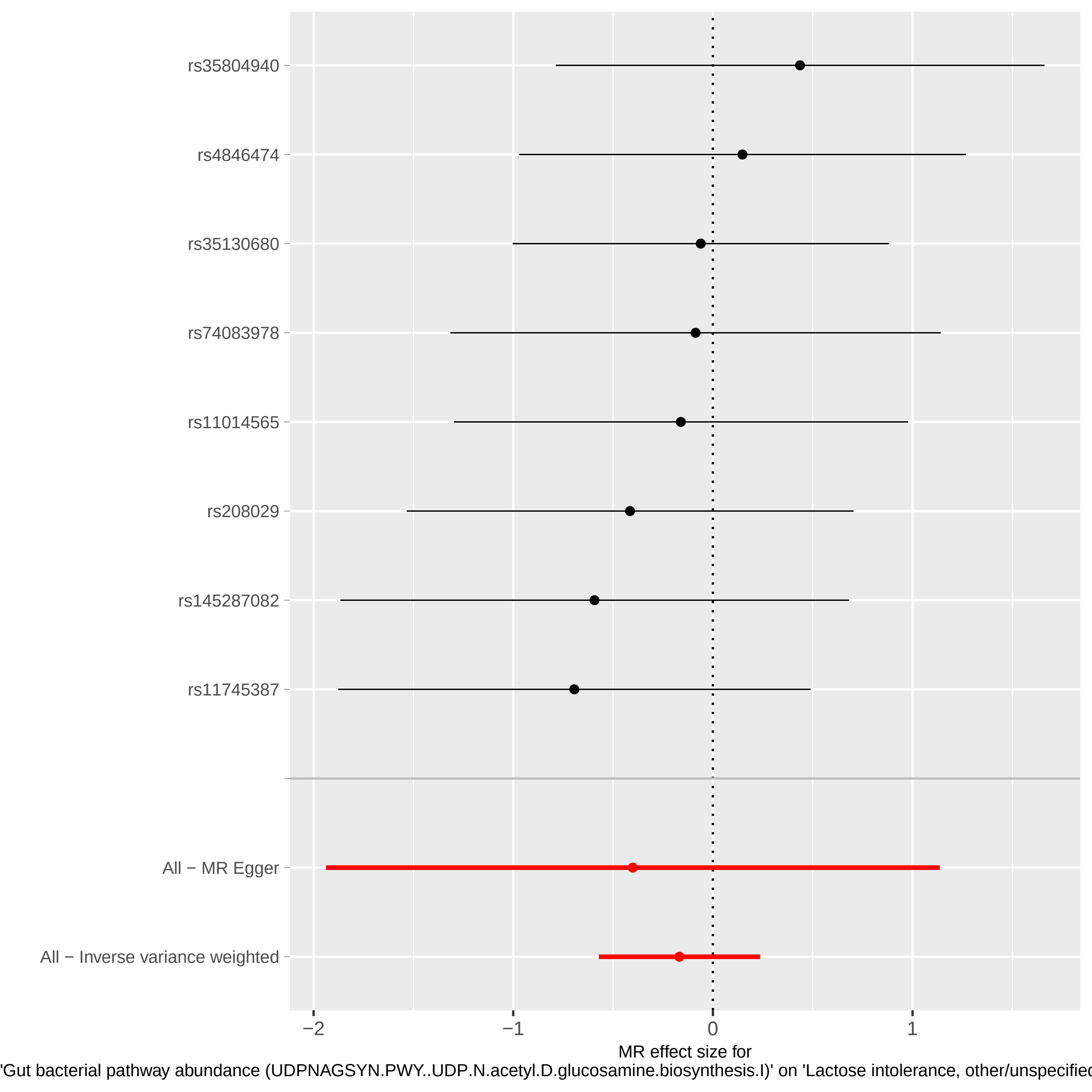

rs35804940
rs4846474
rs35130680
rs74083978
rs11014565
rs208029
rs145287082
rs11745387
All − MR Egger
All − Inverse variance weighted
−2
−1
0
1
MR effect size for
'Gut bacterial pathway abundance (UDPNAGSYN.PWY..UDP.N.acetyl.D.glucosamine.biosynthesis.I)' on 'Lactose intolerance, other/unspecified
